# The Clinical Obesity Maintenance Model: A Theoretical Framework for Bariatric Psychology

**DOI:** 10.3389/fendo.2020.00563

**Published:** 2020-08-14

**Authors:** Jayanthi Raman, Dean Spirou, Lisbeth Jahren, Trine Tetlie Eik-Nes

**Affiliations:** ^1^Discipline of Clinical Psychology, Graduate School of Health, University of Technology Sydney, Sydney, NSW, Australia; ^2^Library Section for Medicine and Health Sciences, NTNU University Library, NTNU–Norwegian University of Science and Technology, Trondheim, Norway; ^3^Department of Neuromedicine and Movement Science, Faculty of Medicine and Health Sciences, NTNU–Norwegian University of Science and Technology, Trondheim, Norway

**Keywords:** obesity, bariatric surgery, disordered eating, executive function, depression, health literacy, emotion dysregulation, habitual cluster behaviors

## Abstract

Ranked highly in its association with serious medical comorbidities, obesity, a rapidly growing epidemic worldwide, poses a significant socio-economic burden. While bariatric procedures offer the most efficacious treatment for weight loss, a subset of patients risk weight recidivism. Due to the heterogeneity of obesity, it is likely that there are phenotypes or sub-groups of patients that require evidence-based psychological support to produce more sustainable outcomes. So far, however, characteristics of patients have not led to a personalized treatment algorithm for bariatric surgery. Maintenance of weight loss following bariatric surgery requires long-term modification of eating behaviors and physical activity. A recent Clinical Obesity Maintenance Model (COMM) proposed a conceptual framework of salient constructs, including the role of habit, behavioral clusters, emotion dysregulation, mood, health literacy, and executive function as interconnected drivers of obesity maintaining behaviors relevant to the field of bariatric psychology. The primary aim of this concise review is to bring together emerging findings from experimental and epidemiological studies relating to the COMM constructs that may inform the assessment and follow up of bariatric surgery. We also aim to explain the phenotypes that need to be understood and screened prior to bariatric surgery to enable better pre-surgery intervention and optimum post-surgery response.

## Introduction

Associated with serious medical comorbidities, obesity is a major risk factor for preventable mortality and morbidity worldwide (1). Bariatric surgery has been shown to be an effective treatment for obesity resulting in greater weight loss than non-surgical treatments (2–4). Although there is a lack of consensus about what constitutes significant weight regain in bariatric surgery studies (5), there is consensus that a subset of patients risk weight recidivism (6–8), and up to 50% of patients experience weight regain within 2 years after surgery (5).

Researchers have suggested that the mechanisms that aid initial weight loss are theoretically distinct from those associated with weight loss maintenance [e.g., (9)]. A growing number of recent studies have indicated that in addition to metabolic and surgical explanations, post-surgical weight regain may be influenced by maladaptive eating, lifestyle behaviors, and psychological co-morbidities (10). Thus, the development of a sound theoretical framework will influence the design of future studies and contribute to greater pre-surgical readiness and improved treatment of post-surgical challenges that impact weight recidivism (11).

**Figure 1:**
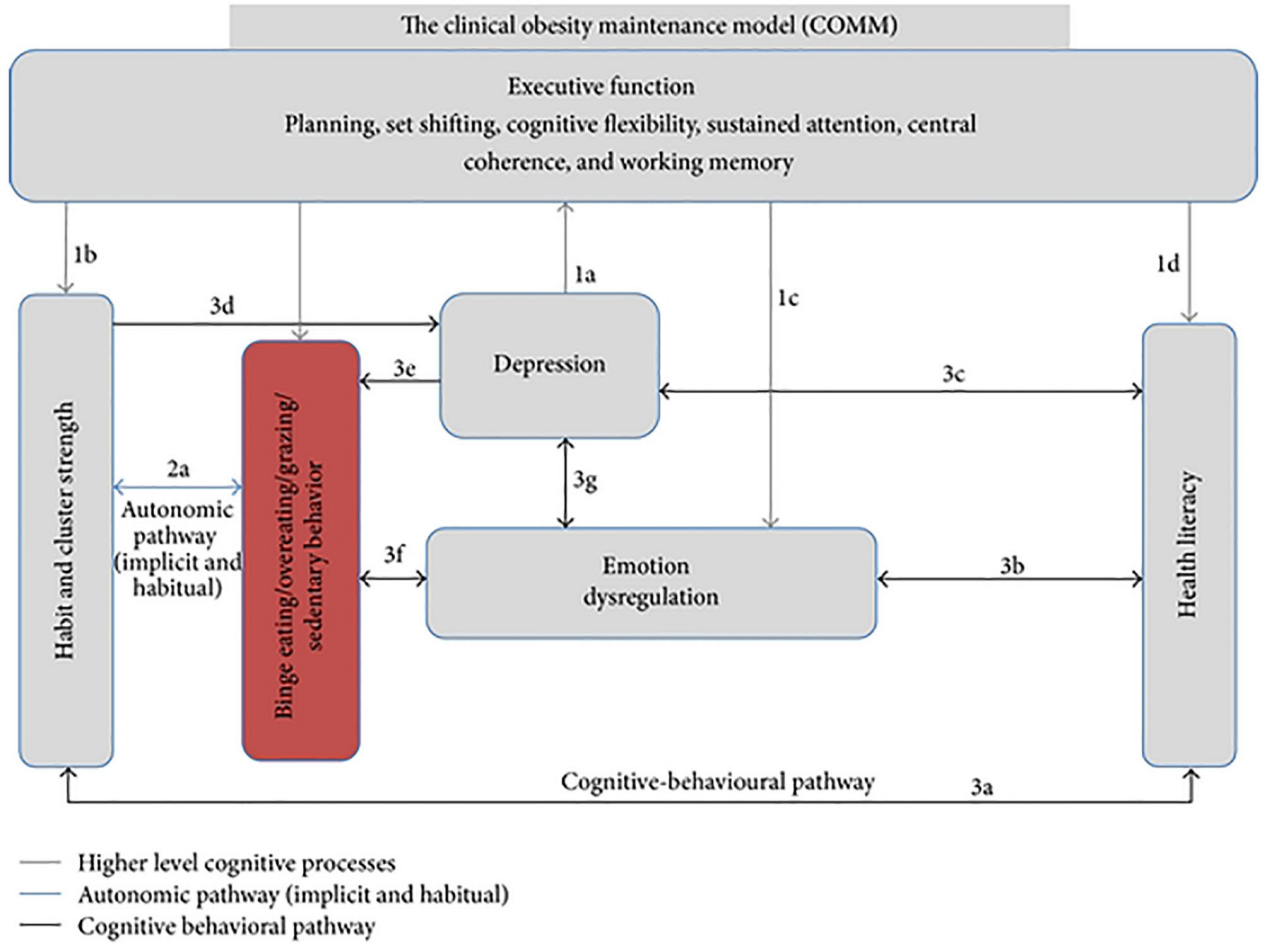
Hypothesized model of the Clinical Obesity Maintenance Model (COMM). The letters indicate direct pathways between variables. From Raman et al. (12). The figure is licensed under Creative Commons Attribution 4.0. License; https://creativecommons.org/licenses/by/4.0.

Incorporating evidence from the fields of eating disorders (ED), neuropsychology, and obesity, the Clinical Obesity Maintenance Model (COMM; 12) has highlighted the need to examine the behavioral and psychological mechanisms that underpin longer-term weight loss maintenance. The specific executive function (EF) deficits at the center of this model [Figure 1; (12)], can be considered to address readiness for bariatric surgery or weight recidivism following surgery. In addition, the COMM suggests that emotion dysregulation, maladaptive habits, behavioral clusters, health literacy (HL), and mood interact with executive functioning and impact eating and physical inactivity leading to maintain obesity (12). In this mini review, we aim to summarize research on the COMM constructs from the past 7 years and provide a theoretical framework of the mechanisms that may be implicated in unsuccessful outcomes following bariatric surgery. Psychological underpinnings associated with post-surgery weight regain will be elucidated and research gaps identified.

## Executive Function

The challenge of weight recidivism has prompted researchers to explore beyond the physiological and psychological aspects of obesity (9, 13). As outlined in the COMM, emerging evidence has suggested that obesity-related disordered eating behaviors (DEB) are linked to deficits in executive functioning (EF) independent of differences in intelligence, level of education, and after controlling for gender, obesity severity, and age (14–18). Although not a unitary construct, EF refers to a set of cognitive processes and behavioral competencies that are involved in initiating and executing strategies, sustaining or flexibly redirecting attention, inhibiting inappropriate behavioral responses, planning, sequencing, and achievement of complex goal-oriented behavior, and cognitive flexibility (19).

A recent meta-analysis found a significant inverse relationship between obesity and EF, including cognitive flexibility, inhibition, decision-making, and planning (17). Nevertheless, the nature by which executive deficits are associated with obesity is unclear. Several plausible explanations have been proposed, including inflammation driven factors, changes in brain-derived neurotrophic factor, dopamine dysregulation implicated in hyperphagia, vascular diseases, neuroendocrine changes, and leptin [e.g., (20–22)]. Each of these factors may independently or collectively contribute to executive deficits in obesity and may influence bariatric surgery outcomes. Furthermore, the direction of this relationship remains unclear. Most studies contend that causality may occur in either direction, with impaired EF increasing the risk of obesity or obesity impacting on EF. For example, a recent systematic review found evidence of a reciprocal influence between obesity and EF (23). Supporting this interpretation, bariatric patients have demonstrated improvements in EF following surgical weight loss (24).

Executive deficits manifested through challenges in self-regulation of eating behaviors may lead to poorer weight loss maintenance after bariatric surgery (25). For example, a study of 37 bariatric surgery patients found that EF was strongly associated with adherence to post-surgical guidelines shortly after surgery (25). Supporting this notion, a recent study has shown that EF predicts a higher body mass index (BMI) 12 months after bariatric surgery (26). Similarly, poorer executive performance 12 weeks post-surgery has been found to be indicative of reduced weight loss at the 3 years follow up (27). Given this body of recent evidence, future research should further explore how best to consider executive deficits in the pre-surgery screening and follow-up of bariatric candidates.

## Disordered Eating Behaviors and Eating Disorders

A substantial evidence-base supports the prevalence of disordered eating behaviors (DEB) and eating disorders (ED) in bariatric surgery outcomes [e.g., (28–31)] with two recent studies finding a higher prevalence of ED in post-surgical bariatric patients with weight regain (32, 33). In addition, Binge Eating Disorder (BED) is highly prevalent among bariatric patients (34). BED refers to eating an excessive amount of food in a discrete period of time, accompanied by a sense of LOC over eating (35). Although gastrointestinal modifications may help to restrict portion sizes post-surgery, new DEB may develop as a compensatory mechanism, with a higher frequency of energy intake (36). In support of this view, recent cross-sectional and longitudinal studies have found evidence on the association between grazing (i.e., intake of smaller portions of food over extended periods) and post-surgical weight regain [e.g., (37)]. Furthermore, researchers have emphasized the role of other DEB, such as emotional eating, night eating syndrome, and picking and nibling in bariatric surgery outcomes (37–39). One longitudinal study found that about 65% of patients with weight regain reported pre-surgery DEB (32). Thus, comprehensive pre and post-surgery assessments and intervention for ED and DEB may hold promise for optimizing bariatric outcomes.

## Depression

An association between depression and obesity has been long supported by clinical and epidemiological studies (40, 41) with a recent meta-analyses providing further evidence of this relationship in bariatric surgery patients (42). A review on the psychological outcomes after bariatric surgery found that pre-surgery depression symptoms reduced at 6, 12, and 24 months after bariatric surgery; however, from 36 months onwards, depression symptoms increased and returned to pre-surgery levels (43). Similarly, a population-based study of 4,793 participants found that bariatric surgery patients had higher levels of depression than others with similar BMI and that initial reductions in depression were not maintained at the follow-up (44). Other studies have also shown that improvements in depressive symptoms following bariatric surgery may not be maintained after the initial post-surgery years (i.e., 1–3 years) and that depressive symptoms may return to baseline or worsen in some patients (45–47).

Post-surgery weight regain and depression may act as a risk factor for one another. Weight regain after surgery has been indicated as a significant risk factor for recurring or elevated depression post-surgery (48, 49). These findings are consistent with the notion that rapid post-surgery weight loss only temporarily aids the remission of depression, which later re-occurs once the surgical benefits decline (43, 50, 51). Similarly, studies have shown that depressed mood is associated with unhealthy lifestyle habits (52), emotional eating, and loss of control (LOC) eating [e.g., (53, 54)]. In particular, post-surgery depressive symptoms have been associated with ED and weight recidivism (42, 55–57). Therefore, the role of depression has important implications for post-surgery functioning and should be monitored and addressed through focused evidence-based interventions.

## Emotion Dysregulation

Individuals with obesity often demonstrate a dysregulated physiological response to intense emotion, known as emotional eating. Specific aspects of emotion dysregulation, such as a lowered tendency to act with emotional awareness (58), difficulty identifying emotions (59), and limited access to emotion regulation strategies (60) have been implicated. A high prevalence of emotion dysregulation in pre-surgery bariatric patients has been shown (61), and pre-surgery maladaptive eating was in one study initiated by both avoidance (of negative affect) and approach (reward sensitivity) behaviors (62). More, emotion dysregulation fully mediated the associations between emotional eating as well as eating in the absence of hunger in another study of bariatric surgery candidates (63). Distress tolerance, an aspect of emotion regulation, has been found in one study to be unrelated to 2-years post-surgical weight loss outcomes but delineated individuals opting for bariatric surgery (64). In contrast, bariatric surgery patients with greater weight loss more frequently applied emotion regulation strategies post-surgery than pre-surgery, compared to patients with lower weight loss (65). Furthermore, there is ample empirical support for a direct link between emotion dysregulation and BED (63, 65, 66), highly prevalent in bariatric patients (30, 34, 43). Accumulating evidence thus indicates that emotional regulatory factors may act as drivers to initiate and/or maintain DEB and ED in bariatric surgery patients. Consequently, future research should further explore the emotional determinants of longer-term weight loss maintenance following bariatric surgery.

## Habit

Habit has been defined as “a process by which a stimulus generates an impulse to act as a result of a learned stimulus-response association” (67). Behaviors driven by habitual automaticity require powerful intentions to override, and developing new habits involves a gradual transfer in cognitive control from purposeful actions to automatic processes (68). Poor dietary choices are likely to be perpetuated by habit (12). For example, habit strength and energy intake were significantly associated in a recent ecological momentary assessment study (69). Furthermore, researchers have asserted that grazing, a DEB described as “mindless,” “distracted,” and “non-anticipated,” should be studied as a habitual and automatic behavior (70). This was supported in a review where a link between grazing and weight recidivism post-bariatric surgery, independent of surgery type and contextual concept of grazing was found (37). In addition, habit has been shown to partially regulate physical activity (71). More, irrespective of the amount of weight loss 6 and 12 months following bariatric surgery, patients may persist with lifestyle habits, such as physical inactivity, lower consumption of protein, fruit, and vegetables, and higher consumption of carbohydrates, sugars, and fats that place them at a high risk for weight regain (72, 73). Maladaptive pre-surgery habits and DEB may thus pose significant challenges to optimal longer-term surgical outcomes (74). Future assessments of bariatric surgery candidates should consider pre-surgery habits as a major driver of weight loss practices (75, 76).

## Health Literacy

Health Literacy (HL) is another construct in the COMM that has been included as a modifiable determinant of obesity maintaining behaviors. HL has been defined as the “cognitive and social skills that determine the motivation and ability of individuals to gain access to, understand, and use information in ways which promote and maintain good health” (77). The COMM considers HL a logical prerequisite to healthy eating behaviors and an active lifestyle. Lower levels of HL are associated with excessive body weight and difficulties overcoming obesity (78) as well as increased use of unhealthy weight loss methods (79), poorer weight loss following bariatric surgery (80), and lower levels of physical activity (81). A patient's HL also contributes to decision-making regarding bariatric surgery. For example, research has found that patients with higher HL and education are more likely to elect bariatric surgery, whereas patients with lower HL and less education are less likely to opt for bariatric surgery (82). Similarly, higher pre-surgical HL is associated with successful weight loss outcomes 12 months after bariatric surgery (83). Evidence also suggests that targeting HL through primary health care interventions results in a significant reduction in weight (84). Therefore, improving one's HL may guide patients to make informed decisions about their treatment and better understand the potential implications of surgery and the barriers to longer-term weight management.

## Behavioral Clusters

Clustering is the co-occurrence of several risk behaviors all of which share an underlying association (85). In obesity, behavioral clusters may interact in multiple ways to maintain the condition, with potentially synergistic effects (86). In support of this view, a study found that bariatric candidates who reported pre-surgical grazing behaviors also reported more alcohol use, less physical activity, and more difficulties in post-surgery lifestyle modification (36). Similarly, longitudinal studies have indicated that pre-surgery problematic alcohol, substance, and tobacco use to be reliable correlates of post-surgery problematic alcohol and substance use in bariatric patients (87–89). Whether these maladaptive clusters are activated as coping and/or compensatory reward mechanisms in a caloric-restricted new lifestyle following bariatric surgery is yet to be explored (90). Similar to previous research that showed smoking, excessive alcohol use, unhealthy eating, and sedentary lifestyle as the salient “big four” modifiable causes of obesity (91), the above findings highlight how pattern recognition of behavioral clusters can inform future research and development of targeted interventions for bariatric surgery candidates. In addition, there is a need for bariatric research to delineate alcohol use disorder from other drug use to help identify behavioral clusters that may further inform post-surgery complications (87).

## Conclusions, Limitations, and Future Research

This mini review aimed to provide a theoretical framework for bariatric psychology by summarizing emerging evidence on the psychological and behavioral constructs incorporated in the COMM. The findings of this review should be considered in light of several limitations. Firstly, a comprehensive delineation on outcome research findings from extant bariatric literature was beyond the scope of this review. For example, the magnitude and rate of weight loss outcomes and other psychological co-morbidities observed post-surgically have previously been shown to differ across types of bariatric surgeries and obesity class (92, 93). Secondly, EF, a central COMM component, has been presented as an overarching construct on account of brevity. Specific aspects of EF, in particular, cognitive flexibility may have important implications for ED and DEB and researchers have called for improved methods in pre-surgery cognitive assessment to help clarify longer term post-surgery outcomes [e.g., (94)]. Similarly, the role of depression in bariatric psychology was discussed in a broader context. Lifetime prevalence rate, type, and severity of depression and associated secondary comorbidities may play an important role in longer-term post-surgery outcomes and need to be carefully assessed at pre-surgery screening (95).

This concise review has outlined key modifiable factors and their putative pathways as interactive drivers of weight recidivism after bariatric surgery. For example, given the established links between EF and DEB, extant studies have begun to incorporate EF, binge eating, and grazing behaviors as outcome measures in the fields of eating behaviors and bariatric psychology [e.g., (36, 37, 96–98)]. Similarly, in a study comparing healthy controls and bariatric surgery patients with and without depression, obesity and depression were shown to have an additive effect on executive performance (99). This highlights the importance of addressing depression and executive deficits before and after bariatric surgery to enhance longer-term surgical outcomes. In addition, the COMM's top-down approach proposes that modification of habits rely on executive processes, which contribute to difficulties in adherence to sustainable eating following bariatric surgery. While executive processes are not typically required to perform habitual behaviors, current research has shown that EF is heavily involved when individuals aim to modify and develop new habits (100).

Under-recognized psychological difficulties and under-treated mental health may negatively impact bariatric surgery outcomes (101). To this effect, weight recidivism-specific HL, specially formulated therapies targeting ED, maladaptive habits and behavioral clusters, specific emotion regulation strategies, and evidence-based psychological therapies for depression may be offered prior to and/or following bariatric surgery. Future research could further explore these associations to gain insight into the determinants of the longer-term efficacy of bariatric surgery. The theoretical framework of COMM should also be further evaluated in bariatric studies, including the use of structural equation modeling and randomized controlled trials.

To conclude, post-surgical weight recidivism is an important public and socio-economic health issue. In addition, regaining weight after bariatric surgery has a significant impact on the patient's mental health and may lead to a recurrence of serious psychological comorbidities. A comprehensive pre-surgical psychological and behavioral assessment partnered with post-surgical management is vital in this regard. As set forth in this review, the underlying mechanisms outlined in the COMM call for a more integrative, multipronged approach in bariatric surgery assessment and care.

## Author Contributions

TE-N and JR conceived the study and were in charge of the overall direction and planning. LJ and DS contributed to the acquisition and screening of data. JR took the lead in writing the manuscript. All authors provided critical feedback and helped shape the review, analysis, and manuscript.

## Conflict of Interest

The authors declare that the research was conducted in the absence of any commercial or financial relationships that could be construed as a potential conflict of interest.
